# Extraintestinal Manifestations of Inflammatory Bowel Disease: A Focus on Kidney Complications

**DOI:** 10.3390/ijms27104614

**Published:** 2026-05-21

**Authors:** Hao Wu, Aiping Lin, Jingshu Chi, Jing Zhang, Bo Peng, Dan Ni, Hong Hao, Zhenguo Liu

**Affiliations:** 1 Department of Gastroenterology, Xiangya Hospital, Central South University, Changsha 410008, China; xiangyawuhao@csu.edu.cn; 2 Division of Cardiovascular Medicine, Department of Internal Medicine, University of Nebraska Medical Center, Omaha, NE 68198-3040, USA; ailin@unmc.edu (A.L.); jchi@unmc.edu (J.C.); jzhang1@unmc.edu (J.Z.); bpeng@unmc.edu (B.P.); dni@unmc.edu (D.N.); hhao@unmc.edu (H.H.)

**Keywords:** inflammatory bowel disease, extraintestinal manifestation, kidney disease, gut–kidney interactions

## Abstract

Inflammatory bowel disease (IBD), comprising Crohn’s disease (CD) and ulcerative colitis (UC), is a chronic relapsing–remitting condition characterized by systemic and intestinal inflammation and immune dysregulation. Up to 47% of IBD patients develop extraintestinal manifestations, yet kidney and urological involvement remain underrecognized. Accumulating evidence has linked IBD to nephrolithiasis, glomerular diseases, tubulointerstitial nephritis, acute kidney injury, chronic kidney disease, and, rarely, amyloid A amyloidosis. Population studies have consistently shown elevated risks for various important kidney disorders in IBD, with CD generally posing a greater risk than UC. The pathogenesis of kidney complications in IBD reflects complex gut–kidney interactions, including metabolic and absorptive abnormalities, shared genetic and immune pathways, intestinal dysbiosis with nephrotoxic microbial metabolites, systemic inflammation, and drug-related nephrotoxicity. Strategies for kidney protection in IBD include increased awareness, close monitoring of kidney function, urinary metabolic profiling in high-risk patients, and prompt nephrology referral for early detection and treatment. Management should include an effective and sustained control of intestinal inflammation, discontinuation of potential nephrotoxic drugs when indicated, and timely diagnosis and treatment of kidney manifestations, as well as integrating kidney complications into IBD guidelines to enhance awareness, ultimately optimizing both the kidney and overall outcomes for IBD patients. Future studies are needed to validate the potential predictive biomarkers for kidney complications and to develop targeted interventions to address shared gut–kidney pathogenic mechanisms in IBD.

## 1. Introduction

Inflammatory bowel disease (IBD), including Crohn’s disease (CD) and ulcerative colitis (UC), is a chronic relapsing–remitting disorder characterized by systemic and intestinal inflammation as well as immune dysregulation [1]. Extraintestinal manifestations (EIMs) are frequently observed in the subjects with IBD and contribute substantially to morbidity, functional impairment, and reduced quality of life for these patients [2]. Indeed, a recent large meta-analysis with over 350,000 IBD patients has shown that at least one of musculoskeletal, dermatologic, or ocular manifestations occurs in 24% of the subjects with IBD, with a notably higher prevalence in CD patients (35%) than in the ones with UC (27%) [3]. Thus, the therapeutic strategies have been rapidly evolving beyond conventional anti-inflammatory and immunosuppressive approaches, with increasing usage of biologics, small-molecule agents, and emerging microbiome- or metabolism-oriented interventions and postbiotics as mitochondrial modulators as summarized in a recent review [4]. These advances highlight the emerging concept on IBD management beyond the control of intestinal inflammation with considerations of preventing and treating systemic and organ-specific complications in IBD.

While musculoskeletal, dermatologic, hepatobiliary, and ocular EIMs are well recognized, kidney and urological complications represent an important yet underappreciated subset of EIMs in IBD. The association between IBD and kidney diseases has been recognized for more than eight decades, with the first report of kidney involvement in 1942 [5]. Since then, large epidemiologic studies have demonstrated that patients with IBD are at increased risk of a wide spectrum of renal complications, including acute kidney injury (AKI), chronic kidney disease (CKD), nephrolithiasis, glomerular diseases (particularly IgA nephropathy), and tubulointerstitial nephritis (TIN) [6,7,8,9,10,11,12,13]. A recent comprehensive meta-analysis including over 860,000 individuals has revealed that IBD patients have 1.59-fold higher odds of developing CKD compared to non-IBD control population [14]. However, the current European Crohn’s and Colitis Organization (ECCO) guidelines and the International Organization for the Study of Inflammatory Bowel Disease (IOIBD) consensus did not specify kidney diseases as an EIM of IBD [15,16]. This may contribute partially to a less desirable level of awareness and/or under-recognition of kidney involvement in IBD patients and, thus, a suboptimal management of these patients clinically. Given the potentially irreversible nature and often-subtle clinical presentation of kidney damage at early stages, heightened awareness, structured screening, and interdisciplinary collaborations between gastroenterologists and nephrologists are desirable for the prevention and/or treatment of kidney diseases in IBD patients.

The present review summarizes and critically appraises the data in the literature on kidney diseases associated with IBD, including epidemiology, clinical spectrum, and potential mechanisms. It also provides evidence-based recommendations for kidney function monitoring and targeted therapeutic interventions for IBD patients, as well as discussions on future research directions in the emerging field of gut–kidney interactions.

## 2. Search Strategy

A comprehensive literature search was primarily conducted in the PubMed database for articles published from 1942 to 2025, with inclusion of the first report on kidney complication in IBD in 1942 [5]. We used the Medical Subject Heading (MeSH) terms and keywords in all possible combinations using Boolean operators with the following search strategies: “inflammatory bowel disease”, “Crohn’s disease”, “ulcerative colitis”, “kidney”, “renal”, “nephrotoxicity”, “renal function”, “kidney function”, “kidney disease”, “renal disease”, “glomerulonephritis”, “interstitial nephritis”, “amyloidosis”, “kidney failure”, “renal failure”. The search was restricted to the articles published in English language. To ensure transparency and reproducibility, the study selection process was conducted based on predefined inclusion and exclusion criteria. Inclusion criteria comprised peer-reviewed original research (including cohort, case–control, and cross-sectional studies), clinical trials, case reports/series, experimental animal studies, and relevant meta-analyses or systematic reviews on the epidemiology, pathogenesis, or clinical management of kidney complications in IBD. Exclusion criteria included unpublished data, conference abstracts without full text, non-English literature, and articles lacking direct relevance to the gut–kidney axis. Regarding the study selection process, the titles and abstracts of the retrieved records were initially screened for relevance by the authors. Subsequently, the full texts of potentially eligible articles were thoroughly reviewed and critically appraised to determine their final inclusions in the present review.

## 3. Epidemiology and Spectrum of Kidney Manifestations in IBD

Kidney manifestations of IBD involve a broad spectrum of kidney disorders with a variety of pathogenesis, clinical presentation, and long-term implications. The most reported kidney conditions associated with IBD include nephrolithiasis, glomerular diseases, TIN, and both acute and chronic kidney injury as summarized in Table 1. Given their distinct epidemiologic patterns and underlying mechanisms, each entity is individually discussed below. When interpreting the results of these population-based studies, several caveats and potential limitations need to be discussed. First, regarding the study designs, much of the cited literature relies on retrospective observational cohorts (e.g., using databases or UK Biobank), which are inherently susceptible to unmeasured or residual confounding conditions. Additionally, reliance on administrative ICD codes may lead to misclassification, often missing a significant number of cases that do not require hospitalization, thereby potentially underestimating the actual prevalence of kidney involvement for these subjects. Second, it could be challenging to assess disease severity. Most database-related studies lack detailed clinical data associated with disease severity, such as endoscopic mucosal appearance, stool frequency, presence of acute dehydration, or malnutrition. Surrogate markers for disease severity, such as the number of IBD hospitalization or surgeries, are also frequently unrecorded. Third, the impact of medication exposure is highly complex. Many studies could not fully specify the names and/or doses of medications, especially for immunosuppressants or biologic agents (e.g., infliximab and vedolizumab).

### 3.1. Nephrolithiasis

Nephrolithiasis is a well-recognized EIM of IBD, with reports of this association dating back to the 1970s [17,18]. Robust epidemiological studies have consistently demonstrated a higher prevalence of kidney stones in the individuals with IBD compared with the general population. While the initial evidence was from several small cohort studies [19,20,21], a comprehensive meta-analysis has estimated the prevalence of kidney stones to be 7.9% in patients with CD and 5.6% in those with UC [22]. More recently, a nationwide Danish cohort study has revealed that patients with IBD have approximately twice the risk of developing urolithiasis compared with non-IBD individuals, with the highest risk observed in CD patients. Furthermore, IBD is associated with a greater likelihood of recurrent urolithiasis events [8]. Although urolithiasis can occur in pediatric patients with IBD, its diagnosis in this population remains relatively uncommon [23].

Urinary metabolic profiles in IBD patients may provide important mechanistic insights: the individuals with nephrolithiasis often have a significantly reduced urine volume with increased excretion of urinary calcium oxalate, which may contribute to kidney stone formation [24,25]. Mendelian randomization analysis further supports a causative relationship between IBD and urolithiasis, with the finding demonstrating that CD increases the risk of stone formation [26]. Thus, IBD, particularly CD, is a significant risk factor for nephrolithiasis.

### 3.2. Glomerular Diseases

Over the past decade, multiple studies have reported a broad spectrum of glomerular pathologies in IBD, including IgA nephropathy, minimal change disease, membranous nephropathy, focal segmental glomerulosclerosis, and, less commonly, membranoproliferative glomerulonephritis [27,28,29,30]. Although evidence regarding specific histopathological subtypes in IBD is derived primarily from small-to-moderate retrospective biopsy study series, these observations have provided important clinical insights. For instance, in a Mayo Clinic study of 111 IBD patients who underwent native kidney biopsy, the most frequent pathological findings were IgA nephropathy (22%), chronic interstitial nephritis (19%), and acute interstitial nephritis (13%) [29]. In contrast, a large Egyptian study that analyzed 896 IBD patients has shown that 218 patients (24.3%) developed significant kidney disease requiring biopsy; among them, amyloidosis is the most common finding (25.5%), followed by IgA nephropathy (16.1%), focal segmental glomerulosclerosis (14.7%), membranous nephropathy (8.25%), and minimal change disease (7.7%) [28].

Population studies have further supported the association between IBD and specific glomerular disorders. One such study has shown that individuals with IBD have nearly twice the risk of developing IgA nephropathy compared with non-IBD controls (HR = 1.96) [11]. Notably, patients with IgA nephropathy have an increased risk of developing IBD both before and after nephropathy diagnosis; and the comorbid IBD in this population is associated with a higher likelihood of progression to end-stage kidney disease [31]. Supporting these findings, a Finnish cohort study has reported an increased prevalence of IBD among IgA nephropathy patients (4.4%) compared with the general population (0.6%) [32]. Additionally, a multicenter retrospective study characterizing IBD-related IgA nephropathy has revealed that most cases are associated with CD (75%) rather than UC (25%) [33]. Collectively, these observations indicate that glomerular disease in the setting of IBD is unlikely to be coincidental, and accumulating data supports a substantive pathogenic link between the two conditions.

### 3.3. Tubulointerstitial Nephritis (TIN)

Whether TIN in patients with IBD is caused by the disease itself or by 5-aminosalicylic acid (5-ASA) therapy remains controversial. Initial limited data from early case reports and small case series suggested a strong association between 5-ASA use and the occurrence of TIN in IBD patients [34,35,36,37,38,39,40,41,42,43,44]. However, other studies have reported that TIN is present prior to the introduction of drug therapy with 5-ASA in some IBD patients or could be the initial presentation of IBD for some subjects without 5-ASA exposure [45,46,47,48,49]. In a 4-year follow-up study of 62 IBD patients receiving 5-ASA therapy, no cases of interstitial nephritis were reported, suggesting that renal impairment might not be associated with 5-ASA treatment [50]. Similarly, in a prospective cohort study with 1529 IBD patients, no difference in 5-ASA consumption was observed between those with and without kidney impairment, supporting the conclusion that TIN is not associated with 5-ASA treatment [51].

However, a large British epidemiologic study of 19,025 IBD patients with 5-ASA usages revealed an increased risk of kidney diseases, which could be partially attributable to the underlying condition of IBD itself. This study also showed that the prevalence of kidney diseases was low and not related to either dose or type of 5-ASA used in IBD patients [52]. Furthermore, the data from population studies has demonstrated that patients with CD have a higher likelihood of developing TIN compared with non-IBD individuals (OR = 1.31) [9]. Collectively, current evidence suggests that, while 5-ASA-induced TIN could occur in IBD patients, TIN is very likely a reflection of primary disease-related kidney manifestation in these subjects rather than drug toxicity. Further studies are needed to establish the direct relationship between TIN and IBD.

### 3.4. Acute Kidney Injury (AKI) and Chronic Kidney Disease (CKD)

AKI and CKD are increasingly recognized as important extraintestinal complications of IBD. Multiple kidney manifestations outlined earlier in the present review such as glomerular pathologies and TIN can precipitate or accelerate the development of AKI and/or CKD in IBD patients. Recurrent urolithiasis and associated repeated interventions due to kidney stones are known risk factors for the development of kidney insufficiency [53,54]. Glomerular diseases, such as IgA nephropathy or focal segmental glomerulosclerosis, can directly impair glomerular filtration capacity, leading to progressive CKD [29,31,55]. Similarly, interstitial nephritis (whether drug-induced or primary immune-mediated) can result in acute deterioration of kidney function and, if unresolved, permanent loss of nephron mass [56].

Beyond these direct kidney pathologies, systemic factors frequently encountered in IBD, such as severe diarrhea, gastrointestinal bleeding, infections, and perioperative complications associated with colectomy, can also precipitate AKI or exacerbate existing CKD [57]. Although early reports of kidney dysfunction in IBD largely described isolated case series [58,59], large population studies have now consistently demonstrated that individuals with IBD have a higher incidence of both AKI and CKD compared with general population [10,12,13]. Pediatric patients with IBD are also vulnerable to CKD, and the risk appears to increase in parallel with the disease severity of IBD [60]. Taken together, AKI and CKD in the context of IBD are typically considered as a reflection of the cumulative impact of IBD-associated renal pathologies, treatment-related nephrotoxicity, and systemic complications, underscoring the importance of identifying and mitigating the potential modifiable risk factors.

## 4. Pathophysiology and Mechanisms for Kidney Manifestations in IBD

**Figure 1:**
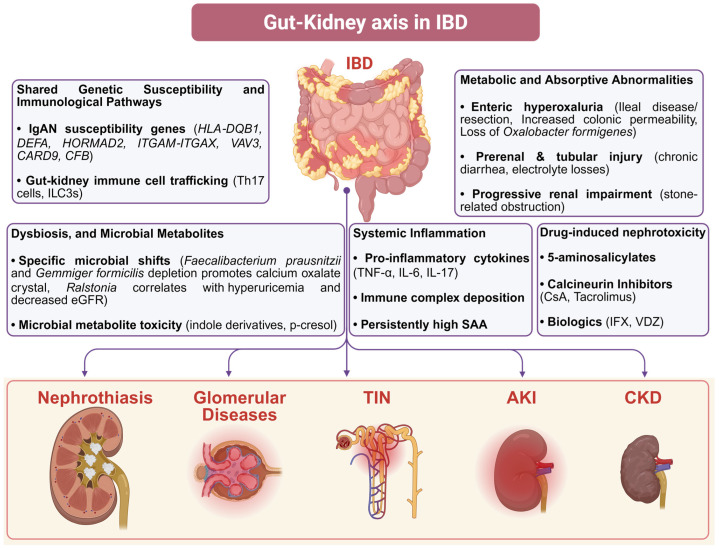
Mechanistic overview of kidney involvement in IBD. IBD-associated kidney complications may arise from several interlinked mechanisms within the gut–kidney axis. Numbers 1–5 denote the primary mechanisms: (1) shared genetic and immune mechanisms; (2) metabolic and absorptive disturbances; (3) gut dysbiosis and accumulation of microbial toxins; (4) systemic inflammation mediated by TNF-α, IL-6, IL-17, and persistently elevated serum amyloid A; and (5) drug-induced nephrotoxicity due to 5-aminosalicylates, calcineurin inhibitors, or biologics. Created in Biorender. Wu, H. (2026) https://BioRender.com/8zv68su.

The gut–kidney axis refers to the functional and mechanistic links between intestinal processes and kidney physiology, mediated through metabolic, immunological, and microbial pathways [61]. In IBD, chronic intestinal inflammation, epithelial barrier disruption, and dysbiosis promote the translocation of microbial products and the generation of gut-derived uremic toxins, which can drive systemic inflammation and oxidative stress [62]. These gut-mediated alterations create a pathogenic milieu that predisposes to kidney complications. This part of the present review summarizes the principal gut-driven mechanistic pathways that may contribute to the kidney pathologies in IBD as shown in Figure 1.

### 4.1. Metabolic and Absorptive Abnormalities

In IBD, chronic mucosal inflammation and surgical interventions, particularly the ones involving the ileum or reconstructive procedures such as ileal pouch–anal anastomosis (IPAA), can profoundly disrupt nutrient and fluid absorption, alter bile salt metabolism, and impair colonic barrier integrity. These alterations may significantly contribute to the key urinary changes, including low pH and decreased urinary volume, that favor uric acid stone formation. Ileal disease or resection further promotes the excessive intestinal absorption of oxalate by increasing oxalate solubility within the lumen and reducing mucosal barrier function [25,63,64,65,66]. This condition, termed enteric hyperoxaluria, arises through three main mechanisms: (1) bile salt malabsorption in diseased or resected distal ileum causes fat malabsorption, allowing the unabsorbed fatty acids binding to luminal calcium. A reduction of free calcium availability limits oxalate–calcium complexation, thus increasing free oxalate absorption and subsequent urinary oxalate excretion and promoting calcium oxalate stone formation. (2) Increased colonic epithelial permeability facilitates oxalate uptake. (3) Loss of *Oxalobacter formigenes*, a commensal bacterium that metabolizes oxalate, reduces intestinal oxalate degradation [67,68]. Oral administration of *Oxalobacter formigenes* has been shown to reduce the levels of oxalate in urine and plasma through intestinal excretion of endogenous oxalate [69]. Severe or persistent hyperoxaluria may lead to chronic TIN [70].

Beyond stone formation, recurrent urinary tract obstructions from calculi can precipitate AKI and, over time, may contribute to the development and progression of CKD in IBD patients. Rarely, penetrating CD involving the urinary tract can cause urinary tract infections, fecaluria, and ureteral strictures. Retroperitoneal fibrosis, sometimes associated with IBD, may lead to secondary obstructive uropathy [71,72,73]. Chronic diarrhea (a common feature of active disease and post-intestinal surgery) promotes persistent volume depletion, leading to pre-renal AKI [74]. Ongoing fluid and electrolyte losses, especially hypokalemia and hypomagnesemia, may significantly impair the tubular function and increase the susceptibility to kidney injuries [75,76]. Collectively, these metabolic and absorptive derangements, due to the combined effects of mucosal injury, surgical alteration, dysbiosis, and chronic diarrhea, create a pathophysiologic environment that strongly predisposes to urinary stone formation, obstructive uropathy, and progressive kidney impairment.

### 4.2. Shared Genetic Susceptibility and Immunological Pathways

Accumulating evidence suggests that IBD and certain kidney complications, notably IgA nephropathy and nephrolithiasis, share some important genetic and immunological underpinnings. Genome-wide association studies have demonstrated the overlapping susceptibility loci between IBD and several EIMs, including ankylosing spondylitis and primary sclerosing cholangitis (PSC). Interestingly, a genetic risk locus that is common to both IBD and kidney stone disease has also been identified, underscoring the presence of systemic pathogenic mechanisms that extend beyond the gastrointestinal tract [77]. Epidemiological data has revealed that IgA nephropathy occurs with higher frequency in patients with IBD compared to the general population [31]. Whole-genome sequencing analyses in IgA nephropathy cohorts have identified multiple susceptibility genes implicated in intestinal barrier regulation, mucosal immune homeostasis, and inflammatory signaling pathways. Key examples include major histocompatibility complex class II (DQ beta 1, also known as HLA-DQB1), defensin alpha (DEFA), HORMA domain-containing protein 2 (HORMAD2), ITGAM-ITGAX, guanine nucleotide exchange factor VAV3 (VAV3), caspase recruitment domain-containing protein 9 (CARD9), and variants in the complement factor B (*CFB*) gene [78,79,80]. These genes are critically involved in some important cell functions such as antigen presentation, antimicrobial peptide production, leukocyte adhesion, intracellular signaling, and complement activation, all central to both intestinal and kidney immune regulations.

Beyond genetic susceptibility, emerging mechanistic data has shown that immune cells primed within the gut can travel to extraintestinal sites through shared homing receptors and chemokine gradients. Bioinformatic profiling has demonstrated a striking similarity in immune infiltration patterns between IBD and IgA nephropathy [81]. Clinically, in patients with PSC-IBD, a substantial portion of gut-primed T lymphocytes have been observed to home to the liver, where they respond to the antigens common to both tissues [82]. Analogously, Th17 cells that are abundant in the gut can exit from the intestine in an S1P receptor-1-dependent manner and subsequently migrate to the kidney via the CCL20/CCR6 axis, contributing to local immune-mediated pathology in the kidney. This gut–kidney trafficking was elegantly visualized using photoconversion in *Kaede* mice with experimental ANCA-associated glomerulonephritis. Furthermore, experimental modulation of the gut microbiota highlights the gut as a pathogenic reservoir for renal inflammation [83]. Likewise, type-3 innate lymphoid cells (ILC3s), traditionally considered as tissue-resident cells within the intestinal mucosa, have been demonstrated to migrate from the gut to the kidney and promote kidney fibrosis. Experimental data reveals that this gut–kidney trafficking is driven by a CXCL16/CXCR6 chemotactic axis initiated by injured kidney tubules. Once localized in the fibrotic kidney, these gut-derived ILC3s utilize PD-1-dependent signaling to amplify interleukin (IL)-17A production, which subsequently activates myofibroblasts directly to drive tissue fibrosis [84]. The homing of gut-derived immune cells to the kidney, leading to kidney injury, highlights a novel and important mechanism for IBD-associated kidney complications. Taken together, the convergence of shared genetic susceptibility loci and recirculating pathogenic lymphoid populations provides a pathophysiologically plausible framework for understanding the heightened incidence of kidney disease in IBD. This perspective could also help develop therapeutic strategies aimed at modulating mucosal immunity to mitigate both intestinal and kidney disease burdens in IBD patients.

### 4.3. Dysbiosis and Microbial Metabolites

Alterations in the gut microbiota are a well-recognized feature of IBD; however, the specific contribution of dysbiosis to IBD-associated kidney injury remains largely underexplored. Although a substantial body of literature links the gut microbial imbalance to kidney injury in other medical conditions in the kidney such as nephrolithiasis, AKI, and CKD, few studies have directly addressed this relationship in the setting of IBD [61,85]. Importantly, given the established roles of pathogenic microbial metabolites and gut-driven systemic inflammation in kidney pathologies, the gut–kidney microbial axis represents an important but currently under-investigated venue for our understanding of kidney complications in IBD.

A range of compositional shifts in the gut microbiota may have kidney relevance in IBD. Both *Faecalibacterium prausnitzii* and *Gemmiger formicilis* are consistently depleted in IBD cohorts; and their absence has been linked to altered bilirubin metabolism. Notably, patients with ileal disease exhibit increased biliary bilirubin concentrations and a higher incidence of gallstones, suggesting a potential interplay between microbial composition and bilirubin handling [86,87]. A recent study has identified the unconjugated bilirubin as a novel regulator of renal calcium oxalate (CaOx) crystal deposition and has proposed that targeting the metabolism of unconjugated bilirubin via the modulation of gut microbiota could be a promising approach to the prevention of CaOx crystal-related nephropathy [88].

Taxonomic associations have also been observed in IBD patients with concurrent CKD. A recent study has shown that the genus *Ralstonia* is significantly enriched in UC patients with CKD as compared to the controls. Moreover, the relative abundance of *Ralstonia* is correlated positively with serum uric acid levels and negatively with estimated glomerular filtration rate (eGFR), implicating a potential role of this taxon in the development of hyperuricemia and progressive kidney dysfunction [89]. Experimental evidence further indicates that gut bacteria may contribute to the initiation and progression of colitis-associated kidney injury, potentially through the mechanisms involving lipopolysaccharide (LPS)-induced amplification of oxidative stress, as demonstrated in the 2,4,6-trinitrobenzene sulfonic acid (TNBS)-induced colitis model [90].

Several gut-derived metabolites, such as indole derivatives, *p*-cresol derivatives, trimethylamine-N-oxide (TMAO), and certain phenyl compounds, are believed to have nephrotoxic effects [91,92,93]. Although their causative role in IBD-associated kidney injury has been minimally investigated, accumulating evidence indicates that these metabolites may have been altered in patients with IBD. For example, the commensal bacterium *Morganella morganii*, isolated from IBD patients, has been shown to produce indole derivatives [94]. Similarly, *p*-cresol can be produced by *Clostridium difficile* and disrupts microbial diversity and compromises the membrane integrity of Gram-negative bacteria. *Clostridium difficile* infection (CDI) is a frequent complication in IBD, and the incidence of patients with CDI concomitant with AKI has been increasing [95,96]. In contrast, recent metabolomic studies reveal that circulating TMAO levels are reduced in patients with IBD, suggesting that TMAO may not play a major pathogenic role in IBD-associated renal injury [97,98]. In general, currently available data suggest that gut dysbiosis and its associated metabolic perturbations, though incompletely defined in the context of IBD, may contribute to kidney injury.

### 4.4. Systemic Inflammation

Systemic inflammation is a hallmark of active IBD and is characterized by elevated levels of circulating pro-inflammatory cytokines such as tumor necrosis factor (TNF)-α, IL-6, and IL-17 [99]. These cytokines not only drive intestinal inflammation but also exert pathogenic effects on distant organs. Previous studies have implicated an important role of the circulating pro-inflammatory mediators in the pathogenesis of several EIMs in IBD, such as venous thromboembolism and osteoporosis [100,101]. Indeed, clinical data has shown that TNF-α antagonists are effective on treating a range of EIMs, such as musculoskeletal, cutaneous, and ocular disorders in IBD patients [102].

Recent experimental studies have provided direct mechanistic links between intestinal inflammation and kidney injury. In murine models, DSS-induced colitis generates a sepsis-like systemic inflammatory response with multiple organ involvement [103]. Activation of Wnt-LRP5/6 signaling in macrophages has been shown to amplify the systemic inflammation in dextran sulfate sodium (DSS)-induced colitis and triggers AKI [104]. In addition, CXCR2-knockout mice are protected against AKI and systemic inflammatory damage associated with DSS-induced colitis, underscoring an important pathogenic role of neutrophil infiltration in gut–kidney inflammatory crosstalk [105]. Additional evidence also implicates immune-complex-mediated kidney injury; for instance, a case series reported the presence of circulating immune complexes in IBD patients with glomerulonephritis, supporting a possible immune-complex-driven mechanism for kidney injuries in IBD [106]. Microphysiological systems designed to recapitulate the gut–kidney axis could serve as an ideal in vitro platform for investigating the mechanisms of kidney involvement in IBD [107]. However, further comprehensive preclinical and clinical studies are necessary to validate these findings.

Poorly controlled chronic intestinal inflammation can also lead to secondary amyloid A (AA) amyloidosis, a rare but severe complication of systemic inflammation [28,108,109]. Persistently elevated serum amyloid A (SAA) during the flares of chronic diseases such as IBD can undergo pathological conformational changes, depositing as insoluble amyloid fibrils in the glomeruli and renal vasculature [110,111]. This process leads to progressive proteinuria, nephrotic syndrome, and subsequent end-stage renal disease. Experimental investigations further suggest that SAA can induce pathogenic Th17 cell responses and promote inflammatory disease progression [112]. Indeed, in patients with IBD, SAA levels are frequently elevated—particularly at the times of active diseases—reflecting the intensity of systemic inflammation and contributing to the pathogenesis of kidney injuries in IBD [113]. Although uncommon, AA amyloidosis carries the risk of high morbidity in IBD patients and underscores the importance of effective and sustained control of systemic inflammation in the prevention of irreversible kidney damage in IBD.

### 4.5. Drug-Induced Nephrotoxicity

Drug-induced nephrotoxicity in IBD is not considered an extraintestinal manifestation per se; however, its discussion is warranted because therapy-related kidney injury can mimic the presentation of kidney involvement as an EIM, making the distinction challenging clinically. In the pre-biologic era, two major classes of IBD therapies were most frequently used: (1) calcineurin inhibitors (cyclosporine and tacrolimus), both of which are well known for their dose-dependent nephrotoxicity, and (2) 5-ASA, with acute interstitial nephritis being the most common clinical presentation. The mechanism for 5-ASA nephrotoxicity remains incompletely elucidated; a genome-wide association study identified a possible link between the HLA region and 5-ASA-induced nephrotoxicity [114], and experimental evidence indicates that 5-ASA can induce mitochondrial dysfunction in renal tubular cells, leading to cellular injury [115]. Currently, a provisional diagnosis of 5-ASA-related nephrotoxicity is typically based on improvement in renal function following drug withdrawal, together with histopathologic confirmation from kidney biopsy.

In the biologic era, overall drug-induced nephrotoxicity remains uncommon; however, sporadic cases have been reported with diverse patterns of kidney injuries, including interstitial nephritis secondary to vedolizumab treatment in CD patients [116,117], as well as infliximab-induced TIN and IgA nephropathy characterized by glomerular deposition of both IgA1 and IgA2 [118,119]. Moreover, biologic therapies have been associated with the development of autoantibodies, lupus-like syndromes, and immune-complex-mediated glomerulonephritis in some IBD patients [120], suggesting that certain biologic-related kidney disorders may be mediated by drug-induced immune dysregulation, leading to de novo autoantibody formation and subsequent immune complex deposition within the kidney tissues.

Clinically, a clear distinction between true IBD-related kidney manifestations and treatment-induced nephrotoxicity (such as 5-ASA-induced TIN) remains a major diagnostic challenge. Even when a kidney biopsy is performed, histopathological features, such as interstitial inflammatory infiltrates, often overlap significantly between primary IBD-driven TIN and drug-associated TIN [29]. Consequently, the differentiation is largely retrospective and heavily relies on the timing of kidney injuries and clinical judgement. The resolution or significant improvement of kidney dysfunction following the prompt cessation of the suspected medication serves as a critical pragmatic indicator of drug-induced nephrotoxicity. Conversely, if renal impairment persists despite medication withdrawal, a true EIM of IBD should be strongly considered.

## 5. Preventive and/or Therapeutic Strategies for Kidney Injuries in IBD

Patients with IBD are at increased risk of kidney-related EIMs and drug-induced nephrotoxicity. Effective management requires early recognition, vigilant monitoring, and coordinated multidisciplinary care involving gastroenterologists and nephrologists.

### 5.1. Monitoring of Kidney Function in IBD

To enhance clinical utility and provide specific clinical recommendations, the existing guidelines, such as the French MONITORED consensus, can be integrated into a risk-stratification framework for kidney function surveillance in IBD patients, tailoring the timing and frequency of monitoring to medication exposure, disease phenotypes, and patients’ clinical features. First, a comprehensive baseline evaluation is mandatory for all IBD patients at the time of IBD diagnosis and prior to initiating new therapies. Subsequently, the monitoring schedule should be framed individually to reflect timing of medication exposure. For patients receiving 5-ASA treatment, reassessment is advised at 1 and 3 months after treatment initiation and every 6 months thereafter, whereas those strictly on biologic therapy without other risk factors for kidney injury can be monitored annually [121]. Second, surveillance must be individualized based on disease phenotypes and clinical features of nephrolithiasis. Notably, compared to patients with UC, those with CD (particularly for the subjects with ileal involvement, prior bowel resection, or chronic diarrhea) may have a profoundly elevated risk of enteric hyperoxaluria and incident nephrolithiasis. For this high-risk cohort or patients with a history of kidney stones, specific urinary metabolic profiling (including pH, calcium, oxalate, citrate, and uric acid) is strongly recommended to detect early lithogenic shifts [122,123]. To ensure timely intervention, such metabolic screening should be performed at least annually along with standard kidney function tests. In addition, comorbid conditions such as hypertension, diabetes, and metabolic syndrome may confer additive risks for CKD progression in IBD, warranting a more frequent monitoring (e.g., bi-annually) [124]. Finally, an effective integration of this risk-stratification monitoring into the comprehensive care plan for IBD patients requires timely and effective cross-disciplinary communications. Nephrology referral is recommended for IBD patients when one or more of the following conditions are present: (1) an increase of ≥30% over the baseline in serum creatinine, (2) a reduction of ≥30% in age-specific eGFR over the baseline, (3) persistent albuminuria/proteinuria, (4) first episode of nephrolithiasis, or (5) new onset microscopic hematuria.

In the pediatric IBD population, kidney function surveillance warrants special clinical consideration due to the unique physiology and dynamics of growth and development. Pathophysiologically, while the major mechanisms for kidney injury, such as immune-mediated glomerular damage, drug toxicity, and enteric hyperoxaluria, largely mirror those in adult IBD patients, the developing kidneys in pediatric patients are extremely vulnerable to these insults, and the underlying genetic predispositions may play a more prominent role in early-onset IBD cases with kidney impairment. Unlike adult patients, evaluation of kidney function in children cannot rely solely on serum creatinine levels, which fluctuate significantly with age, gender, and muscle mass. Instead, pediatric-specific methodologies, such as the bedside Schwartz equation, or Cystatin C-based eGFR calculations should be utilized for accurate kidney function assessment [125]. It is crucial to recognize that even mild, unrecognized kidney impairment in children with IBD can synergize with chronic inflammation and malnutrition, leading to an irreversible growth failure, delayed puberty, and impaired bone accrual [126]. Therefore, while monitoring frequency associated with medication-induced renal toxicity (e.g., for 5-ASA) mirrors that of adult IBD patients, the threshold for clinical interventions must be adjusted for early nephrology referral and optimal management for children with IBD. For pediatric CD patients with extensive disease or bowel resections, screening for nephrolithiasis and enteric hyperoxaluria is equally critical, as early-onset kidney stones often indicate severe metabolic derangements. Ultimately, a low threshold for pediatric nephrology referral is strongly advised for any subject exhibiting an unexplained decline in eGFR, persistent proteinuria, or faltering growth despite optimal IBD control [127].

### 5.2. Treatment of Specific Kidney Manifestations in IBD

In the cases of drug-induced kidney injury in IBD, the most appropriate therapeutic approach is a prompt discontinuation of the offending agent, with consideration of corticosteroid therapy in biopsy-proven acute interstitial nephritis to facilitate the recovery [37,44,116]. For patients with confirmed CKD, selection and dosing of IBD medications require careful adjustment to minimize nephrotoxic risk while maintaining adequate control of intestinal inflammation. The latest practical guide focusing on IBD medications for patients with CKD published in 2025 states that most therapies used in IBD patients, particularly biologic therapies, appear to be safe and effective when used in those with CKD, including those on kidney replacement therapy. However, cautions should be exercised when using conventional therapies and JAK inhibitors in IBD patients [128].

The management of kidney complications directly attributable to IBD itself, including nephrolithiasis, glomerular diseases such as IgA nephropathy, and AA amyloidosis, should begin with the primary goal of achieving a sustained remission of intestinal inflammation. Nephrolithiasis should be managed according to the recommended standard clinical practice guidelines, with specific dietary and pharmacologic considerations tailored to stone compositions and metabolic risk factors. Potential therapeutic interventions for other kidney manifestations are based on very limited data from case reports, small case series, and observational studies. For example, one case report described the successful use of mycophenolate in the treatment of interstitial nephritis in a patient with IBD [129], while another reported that granulocyte/monocyte adsorption therapy effectively controlled interstitial nephritis associated with UC [130]. However, strong evidence from large randomized controlled studies indicates that TNF-α antagonists, which have been shown to be effective on managing other EIMs of IBD in other organ systems such as musculoskeletal tissues, cutaneous, and ocular disorders, may represent a viable therapeutic option for certain glomerular diseases in the context of IBD [102]. Of note, there are some significant concerns regarding the long-term kidney safety for the use of anti-TNF therapies in managing EIMs in IBD patients. A recent large-scale retrospective cohort study involving 10,689 US veterans with incident IBD demonstrated that initiation of TNF inhibitors was independently associated with a 34% higher risk of progressive kidney function decline (defined as a ≥30% reduction in eGFR), compared to the ones without TNF inhibitors, although it did not impact the all-cause mortality [131]. However, the IBD patients receiving anti-TNF therapies typically had more severe, refractory, or extensive intestinal disease. Given that a heightened systemic inflammatory burden is an established driver of kidney impairment, the observed eGFR reduction might largely reflect the natural accelerated decline of the kidney function in the subjects with severe IBD itself, rather than the direct nephrotoxicity of the biologic agents such as TNF inhibitors. Therefore, these findings underscore the complexity of renal outcomes in IBD, highlighting the urgent need for future prospective studies to disentangle the true pathophysiologic impact of the drugs from that of the underlying disease severity on kidney function in IBD.

### 5.3. Ongoing Clinical Trials or Preclinical Studies Targeting Gut-Kidney Interactions

Although current research on IBD-associated kidney complications remains predominantly clinical observational studies with limited mechanistic exploration, the emerging concept of the “gut–kidney axis” in the broad field of nephrology could provide a valuable theoretical framework [132]. Gut microbiome modulation has been emerging as a promising therapeutic strategy. Preclinical evidence indicates that microbial dysbiosis accelerates the production of gut-derived uremic toxins, which exert a direct nephrotoxicity and promote interstitial fibrosis [133]. Consequently, a variety of therapies aimed at restoring microbial homeostasis are under active investigation and/or development for their potential to mitigate systemic inflammation and preserve long-term kidney function. Such interventions include specific probiotics, prebiotics, synbiotics, postbiotics (e.g., short-chain fatty acids such as butyrate), fecal microbiota transplantation (FMT), and phytochemicals (e.g., berberine) [134,135,136,137,138].

Targeting intestinal permeability represents another critical avenue for IBD management. The “leaky gut” phenomenon observed in severe IBD facilitates the systemic translocation of bacterial LPS and inflammatory cytokines, thereby leading to subsequent renal impairment. A couple of preclinical investigations on novel agents targeting intestinal mucosal inflammation, tight-junction modulation, and mucosal healing, have been conducted to restore the intestinal barrier and thereby prevent endotoxemia-induced AKI or CKD progression [139,140].

## 6. Conclusions and Future Directions

Kidney and urological complications of IBD, including nephrolithiasis, glomerular disease, TIN, AKI, CKD, and AA amyloidosis, are increasingly recognized as clinically significant contributors to the overall disease burden and clinical outcomes. The pathogenesis of these kidney conditions reflects a complex interplay of a variety of diverse factors, including (but not limited to): (1) metabolic and absorptive disturbances driven by chronic intestinal inflammation or surgical alterations, (2) shared genetic and immunological pathways, such as trafficking of gut-primed immune cells to the kidney, (3) intestinal dysbiosis and production of nephrotoxic microbial metabolites, (4) systemic inflammatory cascades, and (5) therapy-related nephrotoxicity. Clinically, early recognition, proactive kidney function monitoring, and individualized management that address both intestinal disease control and specific kidney pathologies are critical to preventing irreversible kidney injuries. Multidisciplinary joint management strategies with the collaborative involvement of gastroenterologists and nephrologists should be effectively integrated into the comprehensive care plans for high-risk IBD patients.

Future efforts should focus on: (1) increasing the awareness of kidney involvement in IBD patients and integrating kidney complications into IBD management guidelines, (2) validating the potential predictive biomarkers for subclinical kidney injuries, (3) developing novel and effective therapeutic strategies that target the shared pathogenic mechanisms within the gut–kidney axis, and (4) identifying and validating new target(s) through comprehensive preclinical and clinical studies, to improve both the kidney-related and overall outcomes for IBD patients.

## Figures and Tables

**Table 1:** Overview of population-based studies on kidney and urological disorders in IBD.

Authors (Year)	Study Design	Sample Size	Key Findings
Park S, et al. (2018) [6]	Retrospective cohort study	CD (*n* = 12,585) UC (*n* = 26,227) Non-IBD (*n* = 116,436)	CD is associated with a significantly higher risk of end-stage kidney disease than controls (HR = 6.33).
Vajravelu R, et al. (2020) [7]	Retrospective cohort study	IBD (*n* = 17,807) Non-IBD (*n* = 63,466)	IBD is associated with increased risk of CKD (HR = 1.41).
Dimke H, et al. (2021) [8]	Retrospective cohort study	IBD (*n* = 75,236) Non-IBD (*n* = 767,403)	IBD patients have a 2-fold increased risk of urolithiasis (HR = 2.27) compared to non-IBD individuals.
Zheng W, et al. (2023) [9]	Retrospective cross-sectional study	CD (*n* = 117,631) and Non-IBD (*n* = 117,631) UC (*n* = 70,428) and Non-IBD (*n* = 70,428)	Patients with CD are more likely to have nephrolithiasis (OR = 2.25), TIN (OR = 1.31), and CKD (OR = 1.28) compared to non-IBD individuals.
Liu M, et al. (2023) [10]	Prospective cohort study	IBD (*n* = 4201) Non-IBD (*n* = 413,101)	IBD is associated with higher risks for CKD (HR = 1.57) and AKI (HR = 1.96).
Nakayama T, et al. (2024) [11]	Retrospective cohort study	IBD (*n* = 18,623) Non-IBD (*n* = 4,292,770)	IBD patients have higher risk of developing IgA nephropathy (HR = 1.96) compared to non-IBD individuals.
Saha MK, et al. (2024) [12]	Retrospective cross-sectional study	IBD (*n* = 57,121) Non-IBD (*n* = 5,518,753)	IBD patients have a higher risk of AKI compared with the general population (OR = 1.27).
Yang Y, et al. (2024) [13]	Retrospective cohort study	IBD (*n* = 10,117) Non-IBD (*n* = 50,585)	IBD is associated with higher risks for nephrolithiasis (HR = 1.69), CKD (HR = 1.24) and AKI (HR = 1.97).

## Data Availability

No new data were created or analyzed in this study. Data sharing is not applicable to this article.

## Abbreviations

The following abbreviations are used in this manuscript:

IBD	Inflammatory bowel disease
CD	Crohn’s disease
UC	Ulcerative colitis
TIN	Tubulointerstitial nephritis
EIMs	Extraintestinal manifestations
AKI	Acute kidney injury
CKD	Chronic kidney disease
IOIBD	International Organization for the Study of Inflammatory Bowel Disease
MeSH	Medical Subject Heading
IPAA	Ileal pouch–anal anastomosis
PSC	Primary sclerosing cholangitis
CFB	Complement factor B
ILC3s	Type-3 innate lymphoid cells
CaOx	Calcium oxalate
eGFR	Estimated glomerular filtration rate
LPS	Lipopolysaccharide
TMAO	Trimethylamine-N-oxide
CDI	Clostridium difficile infection
TNF-α	Tumor necrosis factor-α
IL-6	Interleukin-6
AA	Amyloid A
SAA	Serum amyloid A
